# Nicotinamide Limits Replication of *Mycobacterium tuberculosis* and Bacille Calmette-Guérin Within Macrophages

**DOI:** 10.1093/infdis/jiz541

**Published:** 2019-10-31

**Authors:** Jason D Simmons, Glenna J Peterson, Monica Campo, Jenny Lohmiller, Shawn J Skerrett, Sorin Tunaru, Stefan Offermanns, David R Sherman, Thomas R Hawn

**Affiliations:** 1 TB Research & Training Center, Department of Medicine, University of Washington, Seattle, Washington, USA; 2 Center for Global Infectious Disease Research, Seattle Children’s Research Institute, Seattle, Washington, USA; 3 Division of Pulmonary, Critical Care and Sleep Medicine, Department of Medicine, University of Washington, Seattle, Washington, USA; 4 Department of Pharmacology, Max Planck Institute for Heart and Lung Research, Bad Nauheim, Germany; 5 Department of Microbiology, University of Washington, Seattle, Washington, USA

**Keywords:** nicotinamide, nicotinic acid, niacin, *Mycobacterium tuberculosis*, BCG, host-directed therapy

## Abstract

Novel antimicrobials for treatment of *Mycobacterium tuberculosis* are needed. We hypothesized that nicotinamide (NAM) and nicotinic acid (NA) modulate macrophage function to restrict *M. tuberculosis* replication in addition to their direct antimicrobial properties. Both compounds had modest activity in 7H9 broth, but only NAM inhibited replication in macrophages. Surprisingly, in macrophages NAM and the related compound pyrazinamide restricted growth of bacille Calmette-Guérin but not wild-type *Mycobacterium bovis*, which both lack a functional nicotinamidase/pyrazinamidase (PncA) rendering each strain resistant to these drugs in broth culture. Interestingly, NAM was not active in macrophages infected with a virulent *M. tuberculosis* mutant encoding a deletion in *pncA*. We conclude that the differential activity of NAM and nicotinic acid on infected macrophages suggests host-specific NAM targets rather than PncA-dependent direct antimicrobial properties. These activities are sufficient to restrict attenuated BCG, but not virulent wild-type *M. bovis* or *M. tuberculosis*.

*Mycobacterium tuberculosis* is estimated to infect 23%–32% of the world [1, 2] and is the leading infectious killer [3]. Although antimicrobial therapy is effective, clinicians continue to rely on a multidrug cocktail that is marred by toxicity and the need for lengthy therapy, both of which result in adherence issues and drug resistance. Faced with an anemic pipeline of novel compounds, investigators have made efforts to repurpose existing drugs with host-directed targets as adjuncts to existing therapies [4–7].

Nicotinic acid (NA) and its amide nicotinamide (NAM), collectively termed niacin/vitamin B3, were first used therapeutically in the 1930s to treat pellagra. Soon thereafter, when studied to ameliorate radiation-induced mucositis, NAM was serendipitously discovered to shrink lung infiltrates in patients with tuberculosis undergoing radiation therapy [8], and it showed efficacy in animal models of tuberculosis [9] and leprosy [10]. In the decade that followed, the NAM derivatives isoniazid (INH) and pyrazinamide (PZA) were each found to have activity against *M. tuberculosis*. However, the development NAM as a *M. tuberculosis* therapeutic was abandoned owing to growing concern about its antagonism of INH in patients [11] and in a mouse model [12].

Antagonism of INH activity by NAM is poorly defined, but it may be mechanistically similar to the well-described PZA-mediated INH antagonism, considering the structural similarity of these derivatives [13, 14]. Despite this antagonism, PZA remains essential to early sterilizing activity and shortens the required duration of therapy from >9 months to 6 months [15]. This in vivo potency is further at odds with the relatively modest PZA activity in vitro [16], which requires extremely high doses or acidification of the culture medium. This paradox between in vivo and in vitro potency has led some investigators to suggest that PZA also modulates host functions such as proinflammatory cytokine signaling [17, 18] and potentiation of autophagy [19].

NAM and NA are also known to have pleiotropic activities on host inflammatory and metabolic pathways. These drugs are largely associated with anti-inflammatory effects, including down-regulation of proinflammatory cytokine secretion [20–23], disruption of M1 macrophage differentiation [24], and inhibition of macrophage chemotaxis [20, 24–26], and they have proposed protective effects in animal models of sepsis [21, 26–29]. In the case of NA, these effects may be dependent on expression of hydroxycarboxylic acid receptor 2 (HCA2) on mononuclear phagocytes [30, 31]. Interestingly, virulent *M. tuberculosis* strains are proposed to metabolically reprogram macrophages in an HCA2-dependent mechanism to promote lipid droplet formation [32], although this lipid-laden macrophage phenotype is also proposed to be a host-protective response downstream of interferon-γ signaling [33]. Thus, a direct assessment of whether NAM or NA requires HCA2 activity to restrict intracellular growth is warranted.

Previous work supports a unique and critical role for PZA in sterilizing necrotic lung granulomas in a rabbit model [34] and in humans [35], incentivizing an ongoing search for more potent PZA analogues to shorten treatment durations. Considering some treatment success with NAM in humans and the accumulating evidence that NA and NAM can modulate macrophage function, we hypothesized that these PZA analogues limit *M. tuberculosis* replication through host-specific targets, in addition to their known antimycobacterial properties. We confirmed that both compounds had modest direct antimicrobial activity limiting *M. tuberculosis* growth in broth culture at neutral pH, but only NAM was active within the macrophage. These results suggest a host-specific activity of NAM, which was further supported by experiments using *Mycobacterium bovis* bacille Calmette-Guérin (BCG), which is resistant to NAM and PZA due to a nonfunctional nicotinamidase/pyrazinamidase (PncA) required for activation of both of these prodrugs. Unexpectedly, both NAM and PZA restricted BCG growth in macrophages despite having no direct antimicrobial activity in 7H9 broth. However, this macrophage-specific activity of NAM and PZA was lost in a virulent *M. tuberculosis* mutant lacking a functional amidase (H37Rv-pncAdel), suggesting that any host-directed activities that restrict BCG replication are not sufficient to restrict intracellular *M. tuberculosis*.

## METHODS

### Cell Culture, Reagents, and Mycobacterial Strains

Roswell Park Memorial Institute 1640 medium (RPMI 1640) and Dulbecco modified Eagle medium (DMEM) (Gibco) were supplemented with fetal bovine serum (Atlas Biologicals) to a final concentration of 10% (RPMI-10 and DMEM-10). NAM, NA, PZA (all Sigma-Aldrich), and rifampicin (Fisher Biosciences) were reconstituted as aqueous stocks and stored at −20°C to −80°C as single-use aliquots.

U937 cells (CRL1593) and CHO K1 cells (CCL-61) were obtained from the American Type Culture Collection. Before each assay, U937 cells were differentiated in phorbol 12-myristate 13-acetate (Invitrogen) at 50 ng/mL for 3 days, washed once with 1× phosphate-buffered saline, and rested overnight in RPMI-10 without phorbol 12-myristate 13-acetate before infection.

Human monocyte-derived macrophages (MDMs) were prepared from healthy donors using TRIMA LRS chambers (Bloodworks Northwest), as detailed in the Supplementary Materials. CD14^+^ monocytes were differentiated in RPMI-10 plus 50-ng/mL macrophage colony-stimulating factor for a total of 6 days before infection. Human alveolar macrophages were obtained from healthy donors who underwent bronchoalveolar lavage after providing informed consent, as described in the Supplementary Materials.

The *M. tuberculosis* Erdman strain expressing the *luxCDABE* operon from *Vibrio harveyi* was a gift from Jeffrey S. Cox. Wild-type *M. tuberculosis* H37Rv was originally obtained from Colorado State University (H37RvC). *M. tuberculosis* H37Rv-pncAdel was a gift from Helena I. M. Boshoff [36] and cultured in the presence of 50-μg/mL hygromycin B (Invitrogen). *M. bovis* BCG expressing the fluorophore mCherry (BCG-mCherry) was constructed by transforming BCG Russia with the pCherry3 plasmid (gift from Paul Corral and Tanya Parish) and cultured in the presence of 50-μg/mL hygromycin B. Wild-type *M. bovis* (Karlson and Lessel type strain; American Type Culture Collection 19210) was a gift from Carolyn Wallis. All mycobacterial strains were cultured in complete Middlebrook 7H9-GAT medium supplemented with glycerol (Fisher; 4 mL/L), Middlebrook ADC Growth Supplement (100 mL/L), and Tween 80 (Fisher; 0.05% final), with a resultant pH of 6.6 ± 0.2 that was not adjusted except where indicated. Frozen glycerol stocks of each *M. tuberculosis* strain were thawed and cultured in 50-mL conical tubes on rollers to ensure ≥2 doublings before each infection. About 18–24 hours before each infection, cultures were back-diluted in 7H9-GAT to an optical density (OD) of 0.2–0.4.

### Minimum Inhibitory Concentration Assays

Serial dilutions of working drug stocks (10× to 20×) were prepared in 7H9 medium and then added to a 96-well assay plate (Nunclon Delta; Thermo Scientific) containing 7H9 medium to achieve the desired final concentration. *M. tuberculosis* culture was diluted to 0.05 in 7H9-GAT and then added to assay plates at a 1:10 dilution (final OD, approximately 0.005). For *M. tuberculosis*–lux, luminescence reads were recorded daily, as described below. For BCG-mCherry, fluorescence was recorded daily using an Envision multimode plate reader (Perkin-Elmer), with excitation and emission filters of 531 nm and 615 nm, respectively. For wild-type H37RvC and H37Rv-pncAdel, cells were incubated for 4–5 days, and 20 μL of each sample was assayed using the BacTiter-Glo assay (Promega), according to the manufacturer’s instructions. Where indicated, wild-type H37RvC and H37Rv-pncAdel assays were cultured in 7H9-GAT medium adjusted with hydrochloric acid to a pH of 6.0, using an Accumet AB electrode pH meter (Fisher Scientific).

### Intracellular Replication Assay

U937 cells and human MDMs were differentiated as described above and plated on 96-well clear-bottom polystyrene luminometry plates (Corning) or Nunc fluorescence plates (Thermo). Serial dilutions of each drug were added to cells 1 hour before each infection. Actively growing *M. tuberculosis* or BCG-mCherry cultures (OD, 0.3–0.6) were then applied to a 5-μm syringe filter (Millex-SV; Millipore), after which OD was again used to calculate an approximate multiplicity of infection, using the conversion from an OD of 1.0 to approximately 10^8^ colony-forming units (CFUs)/mL, determined experimentally. The inoculum was prepared in RPMI-10 medium and applied to cells, which were centrifuged at 500*g* for 5 minutes and incubated for 4 hours at 37°C. Supernatants were then aspirated, and unbound bacteria were washed twice with warm 1× phosphate-buffered saline before RPMI-10 containing the desired final drug concentrations was replaced. Luminescence was recorded daily (in relative light units), using a Synergy H4 multimode microplate reader (Biotek Instruments). Linear correlation between luminescence in relative light units and CFU counts has been demonstrated using this assay [37].

For CFU assays, MDMs or U937 cells were infected for 4 hours and washed twice, as described above, and then lysed (day 0 samples) with 0.1% Triton X-100 (Sigma) 5 minutes at room temperature. Serial dilutions of each lysate were plated on Middlebrook 7H10 Agar and incubated at 37°C. CFUs were counted between day 14 and day 21. Lysates at the indicated time points were prepared and plated similarly, except monolayers were not washed to avoid cell loss. For enumeration of BCG and *M. bovis* CFUs, supernatants at day 7 were pelleted and combined with each macrophage lysate before serial dilution, although the observed cytopathic effect and presence of extracellular bacterial clumps by microscopy was minimal at these time points.

### Cytokine Responses

An actively growing *M. tuberculosis*–lux culture (OD, 0.3–0.6) was pelleted at 3500*g* for 5 minutes, washed twice, and resuspended in Sauton’s medium. An appropriate volume of this inoculum was then applied to differentiated human MDMs using the OD-to-CFU/mL conversion factor above. For these experiments, monolayers were not washed, and supernatants were simply harvested after 24 hours and filtered using a MultiScreenHTS 0.22-μm plate manifold (Millipore) before removal from a biosafety level 3 facility. Cytokine concentrations were assayed by DuoSet enzyme-linked immunosorbent assay (R&D Systems) using 3, 3′, 5, 5′ - tetramethylbenzidine (TMB) peroxidase substrate (SeraCare) and read on a SpectraMax-Plus plate reader (Molecular Devices).

### Calcium Mobilization Assay for HCA2 Signaling

HCA2 signaling was assessed using an aequorin calcium mobilization assay modified from that described elsewhere [38], with details provided in the Supplementary Materials. CHO-K1 cells transiently transfected with pcDNA3-FLAG-hGPR109a (human HCA2/G protein-coupled receptor 109a), pC15 (Gα15 subunit,) and pG5A (green fluorescent protein–aequorin) were harvested, incubated with 5-μmol/L coelenterazine-h (Promega), and then injected into wells of a 96-well luminometry plate containing 2× concentrations of each drug. Luminescence over 60 seconds was recorded using an Envision multimode plate reader (Perkin-Elmer). Luminesence × time curves were plotted, area under the curve (AUC) values were then calculated and fit to dose titration curves by nonlinear regression to determine half-maximal effective concentrations (EC50) using Prism software (GraphPad Software; 2016). NAM antagonism was performed equivalently, except that all wells contained a fixed dose of NA (2 or 30 μmol/L) and varying doses of NAM to construct inhibition curves.

### CRISPR/Cas9 HCA2 Gene Editing

Details of clustered regularly interspaced short palindromic repeats (CRISPR)/CRISPR associated protein 9 (Cas9) editing of human HCA2 are provided in the Supplementary Materials. Briefly, sense and antisense oligos of the sequence AAAGGACGAAACACCG*CACTAGCCGCACTCATGAAT*GTTTTAGAGCTAGAAATAGCAAG encoding the guide RNA (20 mer) (italics), HCA2 translation start site (underlining), and vector flanking sequences were annealed and cloned into the lentivirus vectors pRRL-Cas9-Puro and pRRL-Cas9-blasticidin for packaging (gift from Daniel Stetson). U937 cells were transduced, and stable lines were selected using puromycin (3 μg/mL) or blasticidin (10 μg/mL). Restriction fragment length polymorphism analysis of a polymerase chain reaction amplicon spanning the HCA2 translation start site was performed using BspHI digest (New England Biolabs), but it revealed a mixed wild-type and edited population (data not shown). This pool of cells was then used to select single-cell clones by limiting dilution, each of which was expanded and screened by restriction fragment length polymorphism and by sequencing to confirm disruption of the ATG translation start site.

### Statistical Analyses

The means of technical replicates were compared by means of unpaired, 2-tailed Student *t* test, using Prism software (GraphPad Software, version 7.0a; 2016), with levels of significance (*P* values) indicated in each figure legend.

## RESULTS

### Differential Effects of NAM and NA on Growth of M. tuberculosis in macrophages and in Broth Culture

**Figure 1.**
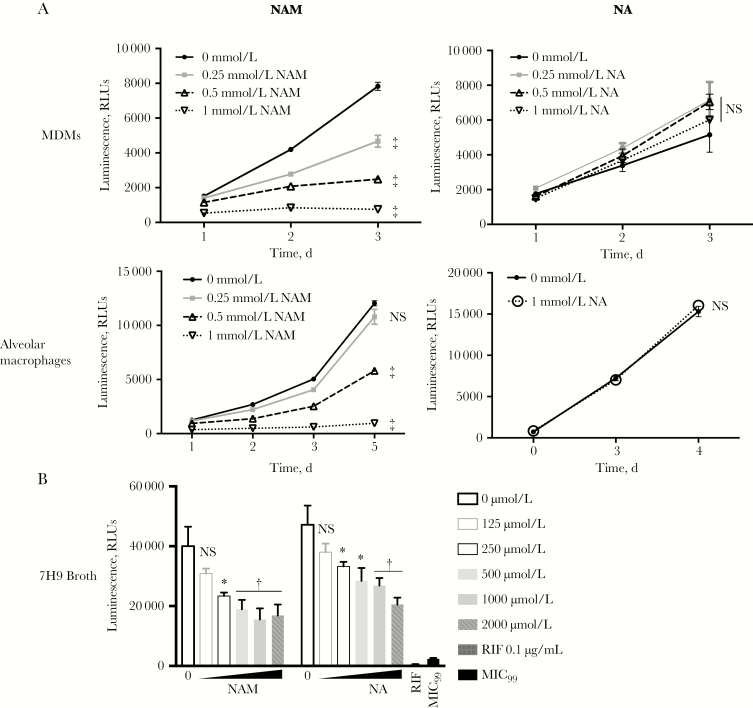
Nicotinamide (NAM) and not nicotinic acid (NA) inhibits intracellular *Mycobacterium tuberculosis* growth within monocyte-derived macrophages (MDMs) and alveolar macrophages. Replication of *M. tuberculosis*–lux encoding the *luxCDABE* operon from *Vibrio harveyi* in MDMs (*A*), alveolar macrophages (*B*), and 7H9 broth culture (*C*) was measured as luminescence. MDMs and alveolar macrophages were pretreated with the indicated doses of NAM and NA for 1 hour before infection at a multiplicity of infection of 0.5–1.0 for 4 hours in the presence of drug. Monolayers were then washed, and medium was replaced with the indicated drug concentrations, after which daily luminescence was recorded. For direct minimum inhibitory concentration determination, 7H9 medium containing the indicated doses of NAM and NA was inoculated with *M. tuberculosis*–lux at a final optical density at 600 nm of 0.005 in triplicate and then incubated at 37°C for 4 days when luminescence was recorded. The 99% minimum inhibitory concentration (MIC_99_) reflects the luminescence of culture containing 1% of the starting inoculum incubated in the absence of drug. Means of 5–6 (*A, B*) or 3 (*C*) technical replicates are plotted, along with standard deviations (*error bars*). *P < .05; †*P* < .01; ‡*P* < .001. Abbreviations: NS, not significant (*P* ≥ .05); RIF, rifampin; RLUs, relative light units.

Previous studies suggest that NAM limits intracellular *M. tuberculosis* growth within human MDMs [39, 40]. To determine whether the derivative NA has similar activity, we treated MDMs from healthy donors with NAM or NA, before infection with *M. tuberculosis*–lux (*M. tuberculosis* Erdman strain encoding the *luxCDABE* operon). The NAM-treated MDMs showed dose-dependent inhibition of *M. tuberculosis* growth (≥250 μmol/L), but, strikingly, no *M. tuberculosis* growth inhibition was observed at all NA concentrations tested (Figure 1A). A direct inhibitory effect on the luminescence chemistry that is unique to NAM is unlikely, because we found similar inhibition of intracellular *M. tuberculosis* (H37Rv) growth, as measured by CFU assay (Supplementary Figure 1).

We next sought to confirm the differential NAM and NA effect on *M. tuberculosis* growth in alveolar macrophages, a cell type central to early recognition of *M. tuberculosis* with immunometabolic activities that may be distinct from those of circulating macrophages [41, 42]. NAM treatment of alveolar macrophages isolated from healthy donors also inhibited *M. tuberculosis* growth at doses ≥ 500 μmol/L similar to the effect observed in MDMs, whereas NA (1 mmol/L) had no effect on *M. tuberculosis* growth in alveolar macropahges (Figure 1A).

We then evaluated the direct antimycobacterial activities of NAM and NA on *M. tuberculosis*–lux cultured in nonacidified 7H9 broth (pH, approximately 6.6). Although neither NAM nor NA demonstrated 90% minimum inhibitory concentration at concentrations up to 2 mmol/L (Figure 1B), each had partial activity, with 50% minimum inhibitory concentration values slightly lower for NAM than for NA (250 and 500 μmol/L, respectively). Importantly, wheras NAM concentrations ≥ 500 μmol/L resulted in dramatic *M. tuberculosis* growth restriction in macrophages, there was minimal additional growth restriction at similar concentrations in 7H9 broth. These results indicate that, in addition to the modest direct antimicrobial activities of NAM and NA, only NAM has enhanced activity within macrophages, possibly through host-specific targets.

### Modulation of Macrophage Cytokine Secretion by NAM and NA

**Figure 2.**
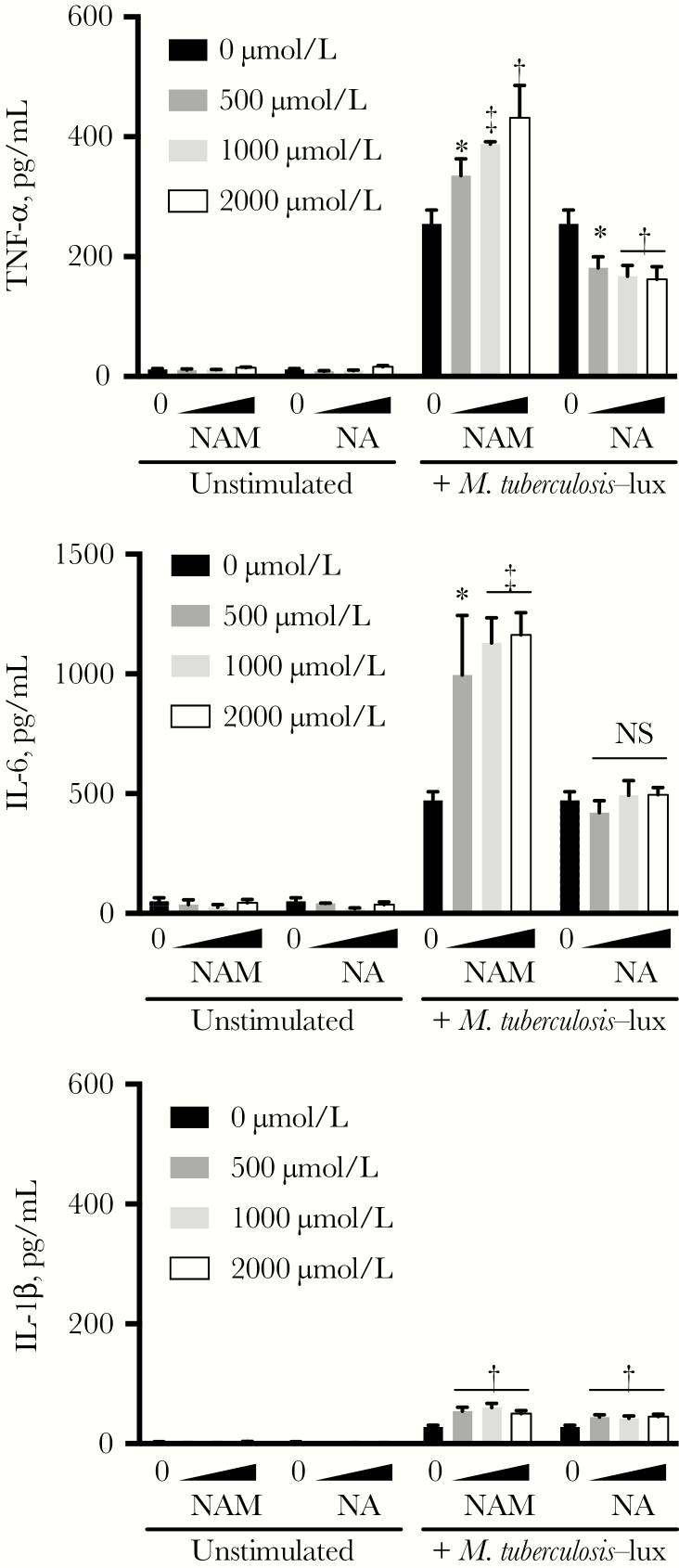
Nicotinamide (NAM) and not nicotinic acid (NA) potentiates *Mycobacterium tuberculosis*–induced tumor necrosis factor (TNF) α and interleukin 6 (IL-6). Monocyte-derived macrophages were pretreated with the indicated drug concentrations for 1 hour before stimulation with *M. tuberculosis*–lux (multiplicity of infection, approximately 10). Cultures were incubated in the presence of drug for 24 hours, after which supernatants were collected for cytokine quantitation by means of enzyme-linked immunosorbent assay. Mean values of 3 technical replicates are plotted with standard deviations (*error bars*). Similar findings were seen in 3 independent experiments. *P < .05; †*P* < .01; ‡*P* < .001. Abbreviations: IL-1β, interleukin 1β; NS, not significant (*P* ≥ .05).

To evaluate whether the disparate activities of NAM and NA in macrophages correlated with differential cytokine responses, we next infected human MDMs with *M. tuberculosis* and quantitated cytokine secretion in supernatants harvested at 24 hours. NA treatment resulted in slight inhibition of tumor necrosis factor α and had no effect on interleukin 6 secretion, whereas NAM potentiated release of both cytokines in a dose-dependent fashion (Figure 2). Although interleukin 1β release was slightly potentiated by each drug, this effect was minimal given low overall stimulation of this cytokine after *M. tuberculosis* infection.

### Dependence of HCA2 on Macrophage Restriction of M. tuberculosis after NAM Treatment

**Figure 3.**
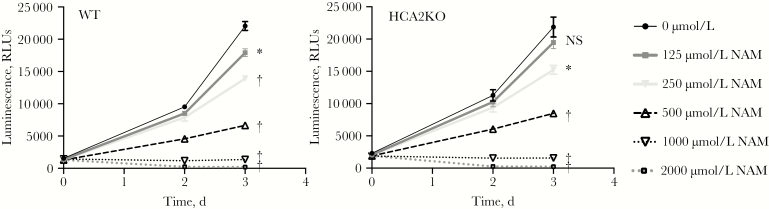
Inhibition of intracellular *Mycobacterium tuberculosis* replication by nicotinamide (NAM) is independent of hydroxycarboxylic acid receptor 2 (HCA2) expression. U937 cells deficient in the G protein-coupled receptor HCA2 were generated by editing the translation start site using clustered regularly interspaced short palindromic repeats (CRISPR)/CRISPR associated protein 9 (Cas9); then single cell clones were expanded after limiting dilution. Inhibition of *M. tuberculosis*–lux replication in wild-type (WT) U937 cells was compared with that in HCA2KO cells in the presence of the indicated doses of NAM, using the macrophage replication assay described for Figure 1. Data shown are plotted as described for Figure 1 and are representative of 2 independent experiments using 5 separate HCA2-edited clones. *P < .01; †*P* < .001. Abbreviations: NS, not significant (*P* ≥ .05); RLUs, relative light units.

To evaluate whether the NAM-specific activity on intracellular *M. tuberculosis* growth was correlated with differential effects on HCA2 signaling relative to NA treatment, we used a calcium mobilization assay in HCA2-transfected CHO-K1 cells. The EC_50_ of NA was <1 μmol/L, whereas NAM showed approximately 1000-fold less activity as an HCA2 agonist (EC_50_, approximately 300 μmol/L), similar to published values [38, 43] (Supplementary Figure 2*A*). We found no inhibitory effect on NA-stimulated HCA2 activity at increasing doses of NAM (Supplementary Figure 2*B*). To formally demonstrate that the NAM-mediated intracellular *M. tuberculosis* growth restriction was independent of HCA2 signaling, we generated HCA2 receptor knockouts in the U937 cell line using CRISPR/Cas9. Replication of *M. tuberculosis*–lux was similarly inhibited in control and HCA2-disrupted U937 cells at doses equivalent to inhibitory concentrations in MDMs (ie, ≥250 μmol/L) (Figure 3). These findings confirm that NAM-mediated inhibition of *M. tuberculosis* in macrophages is independent of HCA2 signaling.

### Effects of NAM and PZA on BCG Growth within Macrophages

**Figure 4.**
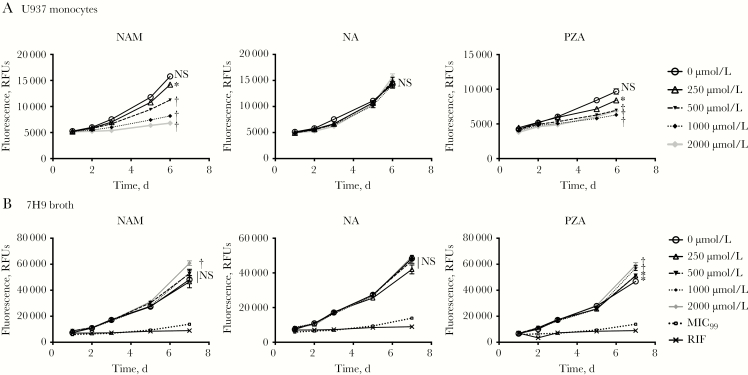
*Mycobacterium bovis*–bacille Calmette-Guérin (BCG) susceptibility to nicotinamide (NAM) and pyrazinamide (PZA) is restored in macrophages. *A,* Phorbol-12-myristate-13-acetate–differentiated U937 cells were infected with a *M. bovis*–BCG fluorescent reporter strain that expresses mCherry (BCG-mCherry). Cells were pretreated and then maintained with the indicated doses of each drug, and fluorescence was monitored. *M.bovis-BCG lacks a functional nicotinamidase/pyrazinamidase (PncA) preventing the activation of prodrugs NAM and PZA*. *B,* To confirm resistance to these drugs, replication of the BCG-mCherry reporter in 7H9 broth in the presence of each drug or rifampin (RIF; 0.1 μg/mL) was similarly measured based on daily fluorescence. Data are represented as means with standard deviations, as in Figure 1. *P < .05; †*P* < .001. Abbreviations: MIC_99_, 99% minimum inhibitory concentration (replication in drug-free 7H9 medium using 1% of the experimental inoculum); NA, nicotinic acid; NS, not significant (*P* ≥ .05); RFUs, relative fluorescence units.

We next evaluated whether NAM also restricts replication of *M. bovis* BCG, which encodes an inactivating point mutation in *pncA* preventing activation of NAM and PZA [44]. NAM inhibited BCG growth at similar concentrations to that seen during *M. tuberculosis* infection in U937 cells (Figure 4A) and MDMs (data not shown), whereas NA treatment had no effect on BCG growth at all doses tested. Surprisingly, PZA also inhibited BCG growth despite the lack of a functioning amidase. However, BCG grew poorly at early time points, especially in MDMs, and thus inhibition by NAM and PZA was most evident at later time points, when cell viability begins to deteriorate and extracellular bacteria can be visualized.

To ensure that the apparent NAM and PZA inhibition was not strictly directed at extracellular organisms, we evaluated BCG growth in 7H9 broth medium under nonacidified conditions (pH, approximately 6.6). As expected, neither NAM nor PZA had direct antimicrobial activity against BCG (Figure 4B). This suggests either that the inhibitory activity of these drugs on BCG in U937 cell culture requires drug activation by host amidases or that these drugs target unknown host cellular processes to restrict mycobacterial growth.

### Correlation of NAM Activity with Virulence in M. bovis and M. tuberculosis strains

**Figure 5.**
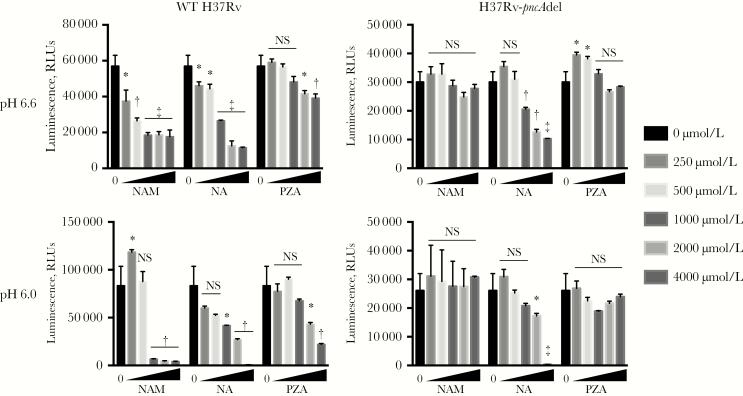
Anti–*Mycobacterium tuberculosis* activity of nicotinamide (NAM) and pyrazinamide (PZA) requires expression of the bacterial *pncA* amidase. Minimum inhibitory concentration assays were performed as described elsewhere in unadjusted (pH, 6.6) and acidified (pH, 6.0) 7H9 medium. Growth of wild-type (WT) H37Rv and a transposon mutant harboring a deletion in the *M. tuberculosis pncA* gene (H37Rv-*pncA*del) was measured on day 5, using the BacTiter-Glo assay (Promega). Means are plotted with standard deviations (*error bars*), with data representative of 3 independent experiments. *P < .05; †*P* < .01; ‡*P* < .001. Abbreviations: NA, nicotinic acid; NS, not significant (*P* ≥ .05); RLUs, relative light units.

**Figure 6.**
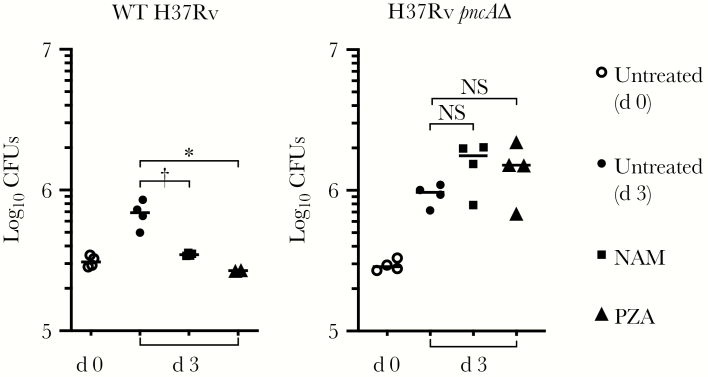
Nicotinamide (NAM) and pyrazinamide (PZA) fail to inhibit *Mycobacterium tuberculosis* replication within macrophages in the absence of the bacterial nicotinamidase/pyrazinamidase (PncA). Monocyte-derived macrophages were infected (estimated multiplicity of infection, approximately 1) with wild-type (WT) H37Rv or H37Rv-*pncA*del for 4 hours in the presence of each drug (1 mmol/L) after which monolayers were washed. Colony-forming units (CFUs) were enumerated after monocyte lysis (0.1% Triton X-100), either immediately (day 0) or after incubation for 3 days in the presence of drug. Mean CFU counts are plotted with standard deviations. No NAM/PZA activity on H37Rv-*pncA*del CFU was observed in 2 independent experiments. *P < .05, †*P* < .01. Abbreviation: NS, not significant (*P* ≥ .05).

We next compared the activities of these compounds on growth of wild-type *M. tuberculosis* (H37Rv) and a mutant harboring a deletion in *pncA* (H37Rv-*pncA*del) [36]. NAM and PZA were active against wild-type H37Rv but not H37Rv-*pncA*del in acidified 7H9 medium, as reported elsewhere [36], whereas NA maintained activity against both strains even under nonacidified conditions (Figure 5). MDMs were next infected with either wild-type H37Rv or H37Rv-*pncA*del in the presence of NAM or PZA (1 mmol/L), and CFUs from lysed macrophages were quantified on days 0 and 3. Both NAM and PZA inhibited wild-type H37Rv growth similarly compared with untreated cultures (Figure 6). However, neither NAM nor PZA showed any activity against H37Rv-*pncA*del within MDM cultures. These findings suggest that if NAM and PZA have host-directed activities in addition to their direct anti–*M. tuberculosis* activity, these activities are inadequate to restrict intracellular *M. tuberculosis* growth or require a functional bacterial PncA.

**Figure 7.**
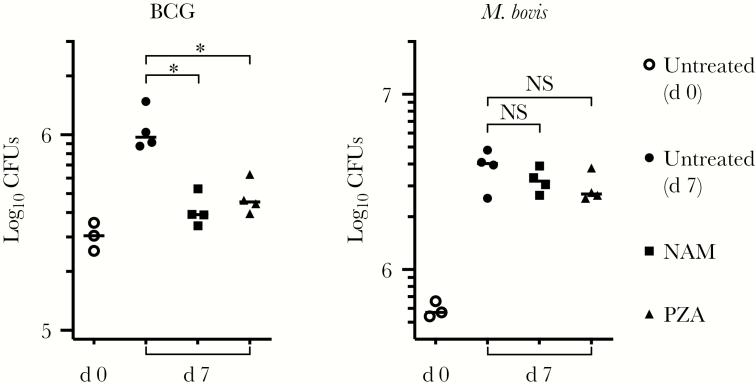
Nicotinamide (NAM) and pyrazinamide (PZA) activity is correlated with Mycobacterium bovis strain virulence rather than the presence of a functional nicotinamidase/pyrazinamidase (PncA). Monocyte-derived macrophages were infected with bacille Calmette-Guérin–Cherry (measured multiplicity of infection [MOI], 2.5) or virulent wild-type *M. bovis* (American Type Culture Collection 19210; measured MOI, 5) for 4 hours in the presence of each drug (1 mmol/L), after which monolayers were washed. Colony-forming units (CFUs) were enumerated from macrophage lysates either immediately (day 0) or after incubation for 7 days in the presence of drug. Mean CFU counts are plotted with standard deviations. Similar results were obtained in 2 independent experiments. *P < .01. Abbreviation: NS, not significant (*P* ≥ .05).

Given that the intracellular growth restriction by NAM and PZA was dependent on PncA for *M. tuberculosis* (Figure 6) but not BCG (Figure 4A), we next evaluated whether these alternative outcomes were correlated with differential virulence. MDMs were infected with either BCG or a wild-type *M. bovis* strain in the presence of each drug. In comparison with untreated macrophages, both NAM and PZA again significantly inhibited BCG growth after 7 days, whereas no inhibition of wild-type *M. bovis* was evident (Figure 7). These findings suggest that the activity of NAM and PZA on *M. bovis* correlates with virulence rather than PncA activity.

## DISCUSSION

Prior studies suggest that NAM, NA, and PZA have host-directed activities in various inflammatory or infection models [18, 20, 21, 27, 29, 31, 45–47]. We hypothesized that these compounds may restrict *M. tuberculosis* replication in macrophages through host-specific targets, in addition to their known direct antimicrobial activities. We found that both NA and NAM have modest direct antimicrobial activity in broth culture, whereas only NAM restricts *M. tuberculosis* growth within MDMs and alveolar macrophages. Direct antimicrobial activity of NAM (and PZA) requires a functioning bacterial PncA amidase, and, accordingly, we confirmed that NAM and PZA had no activity on BCG in broth culture. Surprisingly, both NAM and PZA restricted BCG growth in macrophages but had no activity against either virulent *M. bovis* or *M. tuberculosis* encoding a deletion in *pncA*. Our results are consistent with NAM and PZA having host-directed activities that are sufficient to limit intracellular BCG growth; however, we conclude that these putative host-directed activities are insufficient to restrict intracellular *M. tuberculosis* and *M. bovis* in the absence of a functioning PncA amidase.

Multiple host pathways may be targeted by NAM to restrict BCG growth and contribute to macrophage control of *M. tuberculosis* replication. We found that NAM and not NA potentiated *M. tuberculosis*–induced proinflammatory cytokine secretion, which could contribute to growth restriction. The differential outcomes after NAM versus NA treatment may suggest specific host pathways. Both NA and NAM are biosynthetic precursors to NAD and may affect NAD-dependent host pathways. However, only NAM is expected to inhibit cellular processes that consume NAD as a cosubstrate through end-product inhibition as opposed to processes that use NAD as a redox cofactor. Such processes may include inhibition of sirtuin activity by NAM, PZA, and derivatives like 5-chloro-PZA [48] or inhibition of polyadenosine diphosphate–ribose polymerase family members involved with the host response to other pathogens [45]. Alternatively, end-product inhibition by NAM could disrupt bacteria-encoded enzyme activities, such as tuberculosis necrotizing toxin, known to deplete cellular NAD and result in necrotic cell death [49].

The discordant activities of NAM versus NA may alternatively reflect differential intracellular accumulation of these molecules within macrophages where they have direct antimycobacterial as opposed to host-directed activity. For example, a prior study demonstrated that PZA retained activity in both acidified broth culture and within *M. tuberculosis*–infected macrophages, whereas POA was active only in broth culture [50]. At neutral pH (eg, RPMI macrophage medium), NAM may be readily taken up by macrophages and subsequently converted to the active drug NA (either by host enzymes or by PncA within *M. tuberculosis*), whereas the weak acid NA is mostly deprotonated at neutral pH, which could limit macrophage uptake.

Using the *M. tuberculosis* H37Rv-pncAdel mutant, we failed to confirm our *M. bovis* BCG result, where NAM maintained activity in macrophages in the absence of a functional bacterial amidase. We entertain multiple explanations that warrant further investigation. First, NAM may target host functions that potentiate growth restriction of BCG but are not sufficient to limit *M. tuberculosis*, which encodes additional virulence determinants to enhance intracellular growth. For example, BCG replicates slowly within macrophages and required an assessment of inhibitory activity at later times after infection (ie, >3 days), whereas CFUs from *M. tuberculosis*–infected macrophages were quantitated at earlier times when significant macrophage cell loss was already occurring. Thus, the BCG infection system may be more sensitive in detecting a host-directed activity, but this activity alone is not sufficient to suppress intracellular *M. tuberculosis* growth.

In agreement with this hypothesis, we found that NAM and PZA were no longer active in MDMs infected with wild-type *M. bovis* that encodes additional virulence determinants compared with BCG. It remains to be explored whether these virulence determinants modulate host-protective pathways that are the targets of NAM and PZA. Even if such pathways alone are insufficient to restrict *M. tuberculosis* or wild-type *M. bovis*, they may be important in the context of multidrug therapy.

Taken together, these data suggest that both PZA and its analogue NAM may modulate macrophage functions to restrict intracellular mycobacterial growth in addition to their direct antimicrobial activities. Further investigation is warranted to identify these targetable host pathways, using BCG as a model. Considering the differential potency of PZA in vitro and in vivo, it is plausible that more potent compounds may be identified that modulate these protective host pathways and could serve as adjunctive therapies to reduce the length of antitubercular therapy.

## Supplementary Data

Supplementary materials are available at *The Journal of Infectious Diseases* online. Consisting of data provided by the authors to benefit the reader, the posted materials are not copyedited and are the sole responsibility of the authors, so questions or comments should be addressed to the corresponding author.

jiz541_suppl_Supplementary_Figure_S1

jiz541_suppl_Supplementary_Figure_S2

jiz541_suppl_Supplementary_Material

## Notes

***Acknowledgements.*** We thank Helena I. M. Boshoff for providing the *Mycobacterium tuberculosis*–pncAdel mutant and Jeffrey S. Cox for the *M. tuberculosis*–lux strain. Felicia Nguyen and Brian Lee provided expert technical support with primary cell cultures. We also acknowledge Daniel B. Stetson for expertise and clustered regularly interspaced short palindromic repeats (CRISPR)/CRISPR associated protein 9 (Cas9) reagents used to generate the U937 hydroxycarboxylic acid receptor 2–edited cell line.

***Financial support.*** This work was supported by the Bill & Melinda Gates Foundation (grant OPP1156448 to T. R. H.) and the National Institutes of Health (grants T32AI007044 and K08AI143926 to J. D. S., R01AI093646 to S. J. S., and K24AI137310 to T. R. H.).

***Potential conflicts of interest.*** All authors: No reported conflicts. All authors have submitted the ICMJE Form for Disclosure of Potential Conflicts of Interest. Conflicts that the editors consider relevant to the content of the manuscript have been disclosed.

Presented in part: "Tuberculosis: Translating Scientific Findings for Clinical and Public Health Impact (X7)” (Keystone Symposia on Molecular and Cellular Biology Whistler, British Columbia, Canada, 15–18 April 2018. Abstract 3026.
