# Supplementary figures and images for: Nicotinamide Limits Replication of Mycobacterium tuberculosis and Bacille Calmette-Guérin Within Macrophages
Source: J Infect Dis. 2019 Oct 31;221(6):989–99. doi: 10.1093/infdis/jiz541 (PMC7050990; doi:10.1093/infdis/jiz541)

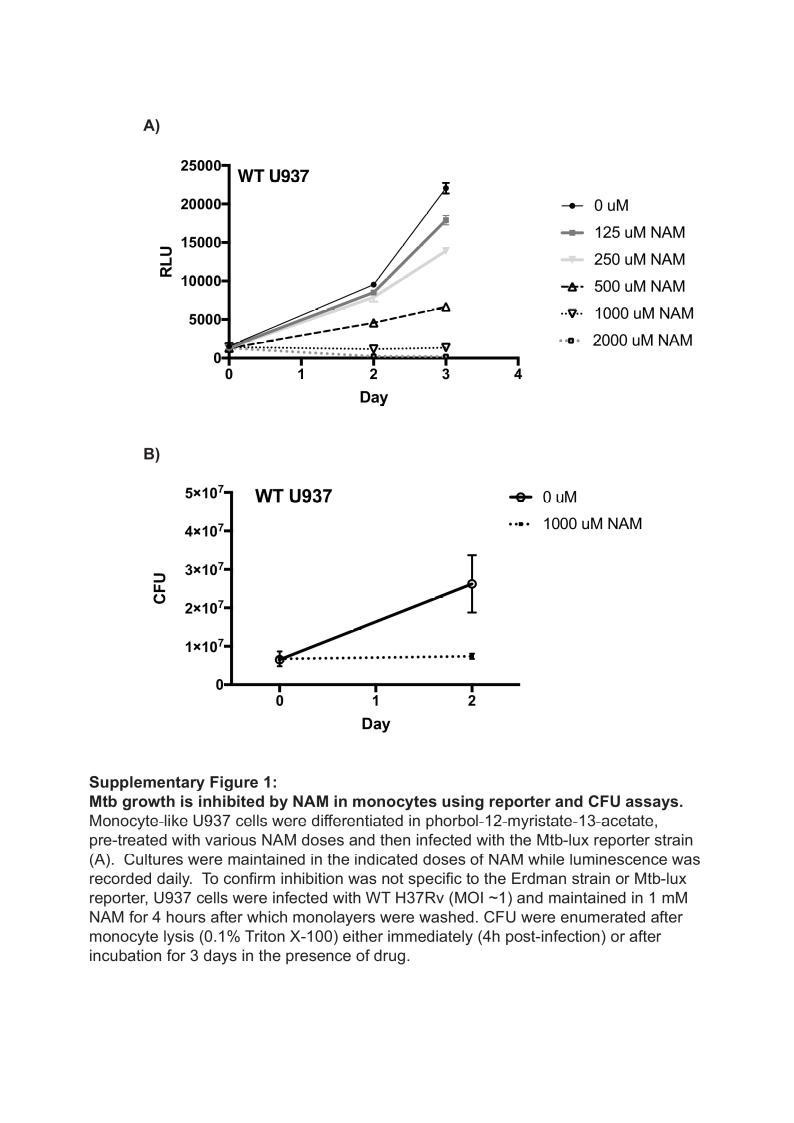

Supplement: jiz541_suppl_Supplementary_Figure_S1 [file jiz541_suppl_supplementary_figure_s1.png]

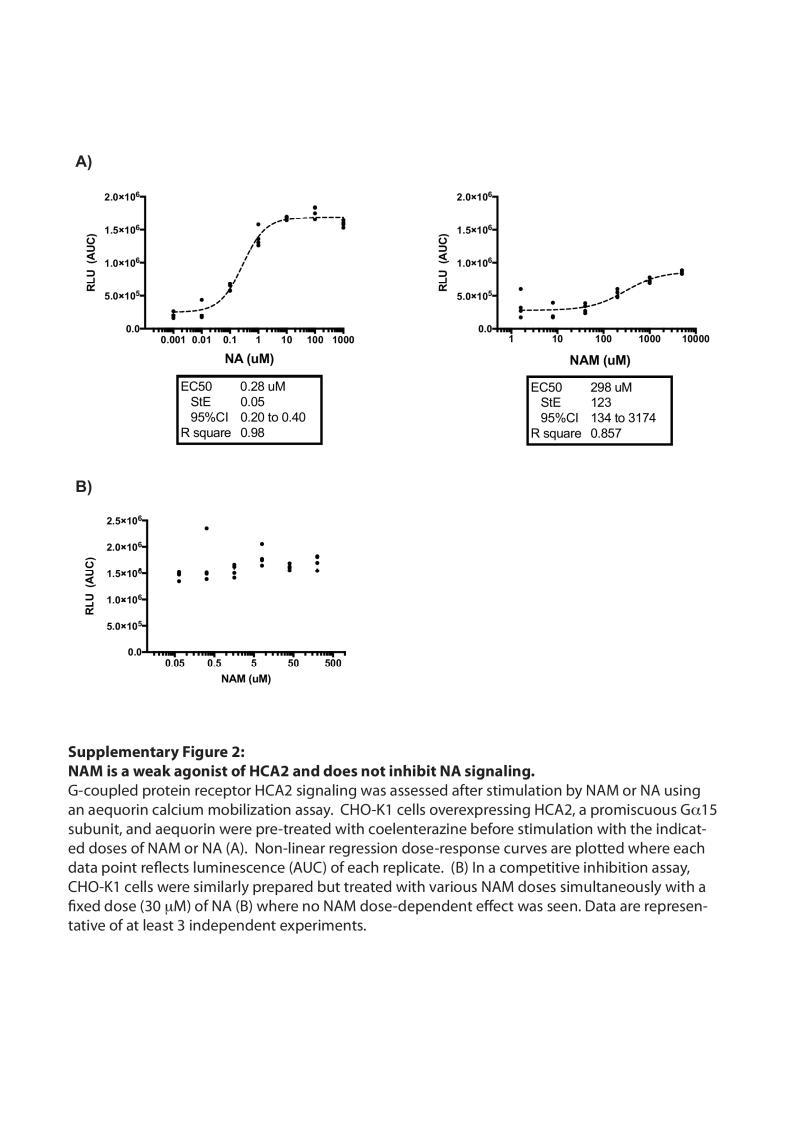

Supplement: jiz541_suppl_Supplementary_Figure_S2 [file jiz541_suppl_supplementary_figure_s2.png]
