# Supplementary material for: Nicotinamide Limits Replication of Mycobacterium tuberculosis and Bacille Calmette-Guérin Within Macrophages
Source: J Infect Dis. 2019 Oct 31;221(6):989–99. doi: 10.1093/infdis/jiz541 (PMC7050990; doi:10.1093/infdis/jiz541)
Supplement: jiz541_suppl_Supplementary_Material [file jiz541_suppl_supplementary_material.docx]

**Supplemental Material**

Supplementary Methods

*Human monocyte-derived and alveolar macrophage preparation*

Human monocyte-derived macrophages (MDMs) were prepared from healthy donors using LRS TRIMA Chambers (Bloodworks Northwest, Seattle, WA) and contents were diluted 15-fold in 1X PBS (Gibco). After Ficoll gradient centrifugation, buffy coats were washed twice in 1X HBSS (Gibco) before quantitation and cryopreservation of peripheral blood mononuclear cells (PBMCs). For each experiment, PBMCs were thawed and resuspended at 2 million cells per mL in RPMI/10 containing 50 ng/mL human M-CSF (Peprotech 300-25) to differentiate for 5 days. Cells were then harvested by scraping and monocytes were isolated by magnetic bead column isolation using CD14 positive selection(Monocyte Isolation Kit I, MACS Miltenyi Biotec). CD14+ MDMs were plated in RPMI/10 + 50 ng/mL M-CSF and rested overnight until infection.

Human alveolar macrophages were obtained from healthy donors who underwent bronchoscopy after providing informed consent. Bronchoalveolar lavage (BAL) fluid was decanted through a 70 μm cell strainer and centrifuged immediately at 4^o^C (300 x g for 10 min). Supernatants were aspirated and the cell pellets were washed twice. Cell pellets were then resuspended in RPMI/10 containing penicillin (100 IU/mL) and streptomycin (100 ug/mL) and purity was assessed after cytospin. Cells were plated on 96-well clear-bottom polystyrene luminometry plates (Corning) at 85,000 cells per well and allowed to adhere for 2 hours. Cells were then washed 6X with warm HBSS (with calcium and magnesium supplementation, Gibco) and rested overnight at 37^o^C prior to infection.

*Calcium mobilization assay for HCA2 signaling*

HCA2 signaling was assessed using an aequorin calcium mobilization assay modified from that previously described[38]. CHO-K1 cells were transiently co-transfected using Fugene HD (Promega) with pcDNA3-FLAG-hGPR109a expressing the human HCA2 receptor, pC15 (pcDNA3 expressing a promiscuous Gα15 subunit from mouse, but competent for human GPCR signaling), and pG5A expressing a GFP-aequorin fusion protein. Media was replaced at 24 hours and then cells were harvested with 0.25% trypsin/EDTA (Gibco) at 48 hours, pelleted and re-suspended in HBSS/+0.1% BSA at ~2 million cells per mL. Cells were then incubated at 37^o^C on an orbital shaker in the presence of 5 μM coelenterazine h (Promega) for 2h, then diluted to ~7.5 x 10^5^ cells per mL in HBSS/BSA immediately before assay. Serial dilutions of drug were prepared at a 2X final concentration in a 96-well Nunclon Delta luminometry plate (Thermo Scientific) and loaded onto an Envision multimode plate reader (Perkin-Elmer) equipped with injectors. Luminometry data were acquired for 10 seconds to establish a baseline, then cells were injected into an equal volume of 2X drug dilution (75 μL, ~50,000 cells) in each well of the assay plate. Data were acquired each second (25 msec per read) for 60 seconds and were exported into Prism (GraphPad Software, Inc. 2016) to calculate AUC for each drug concentration (each performed in 3-4 replicates). To eliminate the artefactual “flash” resulting from transient calcium mobilization immediately upon cell injection that was ligand-independent, data points correlating to seconds 11-13 were removed from all samples. Non-linear regressions were then fit to dose titration curves for EC50 calculation using Prism software. NAM antagonism was performed equivalently except that all wells contained a fixed dose of NA (2 or 30 μM) and varying doses of NAM to construct inhibition curves.

*CRISPR/Cas9 HCA2 gene editing*

Sense and anti-sense oligos of the sequence “AAAGGACGAAACACCG*CACTAGCCGCACTCATGAAT*GTTTTAGAGCTAGAAATAGCAAG” encoding the gRNA 20mer (italics), HCA2 translation start site (underline) and vector flanking sequences were annealed prior to cloning. The lentivirus vectors pRRL-Cas9-Puro and pRRL-Cas9-Blasticidin containing dual promoters to express the gRNA and Cas9 along with the indicated selection marker were double-digested with SbfI and AfeI (New England Biolabs) and used to clone the annealed insert using the In-Fusion technology (Takarabio/Clontech). Lentivirus was packaged in LentiX-293T cells (Takarabio/Clontech) using an equal mixture of these Puromycin and Blasticidin expression vectors along with packaging vectors pRSV-Rev, pMD2.g and pMDLg/pRRE. Lentivirus-containing supernatants were harvested after 48h, passed through a 0.45 μm syringe filter, and then used to transduce U937 cells. Double selection using puromycin (3 μg/mL) and blasticidin (10 μg/mL) was performed at 48h post-transduction for 3 days before media was replaced and edited pools were expanded. RFLP analysis (using BspHI, New England Biolabs) of a PCR amplicon spanning the HCA2 translation start site to which the gRNA was directed (primers TTTCCTGGTAACCATTCAGTCA and CCTTGACAATGAAGTCATCTCG) revealed a mixed WT and edited population (data not shown), so this pool of cells was then used to select single-cell clones by limiting dilution. Once expanded, several of these single-cell clones demonstrated pure edited populations by RFLP and by sequencing where the ATG translation start site was disrupted (including clone D6 shown in Figure 3C). RFLP and sequencing data is available upon request.
